# The association between national income and adult obesity prevalence: Empirical insights into temporal patterns and moderators of the association using 40 years of data across 147 countries

**DOI:** 10.1371/journal.pone.0232236

**Published:** 2020-05-13

**Authors:** Debabrata Talukdar, Satheesh Seenivasan, Adrian J. Cameron, Gary Sacks

**Affiliations:** 1 Community of Global Health Equity and School of Management, State University of New York at Buffalo, Buffalo, New York, United States of America; 2 Department of Marketing, Monash Business School, Monash University, Melbourne, Victoria, Australia; 3 Global Obesity Centre, Deakin University, Geelong, Victoria, Australia; McMaster University, CANADA

## Abstract

**Background:**

At a country level, population obesity prevalence is often associated with economic affluence, reflecting a potential adverse outcome concomitant with economic growth. We estimated the pattern and strength of the empirically observed relationship between national income and adult obesity prevalence, and the moderating role of countries’ macro-environments on this relationship.

**Methods:**

We assembled data on national obesity prevalence, income and a range of variables that characterize macro-environments related to 147 countries from multiple international organizations and databases. We used a Bayesian hierarchical model to estimate the relationship (elasticities) between national income (using Gross Domestic Product Per Capita, GDPPC) and adult obesity prevalence, and the moderating effects of five different dimensions (globalization orientation, demographic characteristics, economic environment, labor market characteristics, and strength of health policies) of countries’ macro-environments on the income elasticities. Using the latest (2019–2024) available national income growth projections from the International Monetary Fund, we forecast future global trends in obesity prevalence.

**Findings:**

Over the 40-years 1975–2014, adult obesity prevalence increased at a declining rate with GDPPC across the 147 countries. The mean income elasticity estimates were 1.23 (95% credible interval 1.04–1.42) for males and 1.01 (0.82–1.18) for females. The elasticities were positively associated with the extent of political globalization and negatively associated with urbanization and share of agriculture in the national GDP. Income based projections indicate that obesity prevalence would continue to grow at an average annual rate of 2.47% across the studied countries during 2019–2024.

**Conclusions:**

Population obesity prevalence exhibits a positive relationship with national income and there is no evidence that the relationship, while weakening, actually turns negative at higher income levels (“obesity Kuznets curve”). Based on current trends, global obesity prevalence will continue to increase during 2019–2024, with the rate of growth higher in low- and middle-income countries. As most people currently live in low- and middle-income countries with rising incomes, our findings underscore the urgent societal imperatives for effective policy initiatives, especially those that target the concomitant “nutrition transition” process with economic affluence, to break or at least further weaken the positive relationship of population obesity prevalence with national income.

## Introduction

Globally, the prevalence of overweight and obesity is high and continues to increase unabated. Pooled analysis of 1698 population-based studies found that the global prevalence of obesity in adult males increased from 3.2% to 10.8% between 1975 and 2014, with a corresponding increase from 6.4% to 14.9% in females [1]. More than 2.1 billion people, or almost 30% of the global population, are overweight or obese, with consequent adverse economic impact estimated to be $2 trillion or 2.8% of annual global GDP [2]. Obesity is one of the top five risk factors for mortality globally, and reducing obesity prevalence has been identified as the most likely strategy to prevent loss of life in modelling to 2040 [3,4]. Given its highly significant health and economic costs, obesity is a clear global public health priority, and understanding the many factors that underpin obesity is of vital importance to advance coordinated public health policy initiatives to combat this epidemic [5,6].

At a national level, economic affluence has been found to have a positive association with obesity [7,8]. While economic growth has, throughout history, been one of the biggest positive influences on public health, the rise in global obesity levels that has accompanied rising global income [5] represents a potential negative externality–a case of a desirable societal goal (economic growth and development) being related to an undesirable societal outcome (increase in obesity and its consequences). Importantly, however, there are substantial differences in national macro-environmental factors that influence obesity-related health outcomes [9]. These factors (e.g., policies [5,9], globalization [10], urbanization [11], female participation in the workforce [12]) can moderate the association between national incomes and obesity levels through many pathways, such as shaping population food consumption and dietary habits [11], lifestyle and physical activity trends [11], and other psychosocial and behavioral effects (e.g., food culture, culture around body size) [5].

Given the clear importance of both economic development and obesity prevalence, it is imperative that we gain an in-depth and systematic understanding of the national income-obesity prevalence relationship and the macro-environmental moderators of this relationship. At an individual level, income is expected to have positive relationship with obesity—as individuals’ incomes increase, they can afford excess food beyond the basic subsistence levels, which leads to higher nutrient intake [8]. Further, an increase in population incomes is typically accompanied by a “nutrition transition” (involving substantial shifts in how food is grown, processed, distributed and consumed) [13], and a simultaneous “physical activity transition” (in which shifts from agrarian to industrial and white-collar occupations result in an increase in physical inactivity) [13,14], which exacerbate the effect of income on individuals’ likelihood of being obese. At an aggregate national level, this in turn may manifest as a positive relationship between national income and obesity prevalence. However, beyond this basic understanding, there is ambiguity around the pattern of this critical relationship.

Specifically, if an increase in individuals’ incomes is associated with a proportional increase in the likelihood of them having obesity, it would result in a linear relationship between national income and obesity prevalence. However, as incomes increase beyond a certain level, individuals’ knowledge about the health benefits of proper weight [15] and their ability to invest in their personal health, for example, through purchasing healthier foods and investing in physical activities (e.g. gym memberships) would increase [8]. This in turn could mitigate or even reverse the effect of individuals’ incomes on their likelihood of being obese. As a result, obesity prevalence at national level could increase at a decreasing rate with national income (reflecting a log-log relationship form), or could even decrease as national incomes increase beyond a certain threshold level, consistent with an inverted-U relationship or an obesity Kuznets curve [8, 15–19]. So, conceptually the relationship between national income and obesity prevalence could take one of these three distinct forms—linear, inverted-U (Kuznets curve) or log-log form. What does actual empirical evidence say about the pattern of this relationship? It still remains unclear from the relevant extant literature because of undernoted limitations of the related studies.

Studies in the extant literature that investigate the relationship between income and obesity have followed two distinct but complementary approaches: one group [20–22] analyzes disaggregated data at the individual person level, and another group [7, 16–19, 23] analyzes aggregated data at the country level. With its common focus on understanding the aggregate level relationship between national income and population obesity prevalence, it is the latter group of studies that serves as the most relevant extant literature for our study. Most of the studies in this latter group essentially use pure cross-sectional data across countries for a specific year. None of these studies estimate how the strength of the relationship between national income and population obesity prevalence differs across the analyzed individual countries. However, given the widespread differences across countries in multiple facets such as body composition, food habits, cultural and environmental factors [5], the longitudinal pattern of the relationship between national income and population obesity prevalence and the relationship strength are likely to vary widely across countries. As such, statistically robust empirical analysis of the pattern and strength of this relationship requires its estimation at country-level using longitudinal data over a large number of years for each individual country, in addition to the cross-sectional data across a large number of countries. Unfortunately, the aforesaid extant literature is conspicuous for the near absence of studies that combine cross-sectional data across a large number of countries with significant longitudinal data for each country to estimate country-level income-obesity prevalence relationship. In addition, although some past studies have investigated the role of national macro-environmental variables on obesity prevalence [10–12,18] per se, none of the existing studies have considered the moderating roles of these variables on the critical relationship between national income and population obesity prevalence.

The goal of our study is to build upon and advance the existing literature on the national income and population obesity relationship by addressing the above noted limitations of the past studies. To that end, we assemble an extensive data set covering 40 years (1975–2014) and 147 countries (accounting for 94.4% of the global population and 95.7% of total global economic output) for an in-depth analysis of the relationship. We use the data to investigate the longitudinal pattern of the income-obesity relationship within individual countries and how the strength of such relationship varies across the analyzed countries. We also investigate several key macro-environmental moderators of the relationship, and finally predict the future trends in obesity prevalence based on projected future national income levels.

## Methods

### Data sources

We assembled data on national income, obesity prevalence as well as a range of related country-specific macro-environmental variables from multiple international organizations and databases. Specifically, the prevalence of obesity (i.e. proportion of population with Body Mass Index or BMI > 30kg/m^2^) [17] among adult males and females was obtained for 147 countries over 40 years (1975–2014) from the World Health Organization’s (WHO) global health observatory database [24]. Obesity prevalence data in the WHO database is age-standardized and is based on NCD risk factor collaborations’ estimates from population-based measurement studies [1]. Annual *Gross Domestic Product Per Capita* (GDPPC) of the studied countries over the same period was sourced from the World Bank, and was used as the measure of national income [25]. We used the latest (2019–2024) available data on projections of income growth in the studied countries from the International Monetary Fund (IMF) to conduct our forecasting analyses of future global trends in obesity prevalence [26].

As for the country-specific macro-environmental variables that are likely to moderate the national income-obesity relationship, our analyses–drawing on the extant literature—focused on the following five major dimensions. First, globalization orientation of a country is likely to influence the nutrition transition process that typically accompanies national income growth. Such influence occurs through increased exposure to global consumption trends, as well as accessibility to highly processed foods (typically high in energy density) from the industrialized world [10]. Second, demographic characteristics like urbanization and share of elderly population are likely to moderate the income-obesity prevalence relationship. Urbanization is usually associated with increased access to unhealthy food and lower physical activity levels, and age is also positively associated with BMI in general [19,20].

Third, economic factors like the share of services in a country’s GDP indicate the extent of population involved in industrialized and white-collar occupations involving lower physical activity, which could accentuate the income-obesity relationship [14]. Fourth, labor market characteristics such as female working hours could have an impact on food preparation at home, healthy eating and physical activity, thereby influencing the income-obesity relationship [16]. Finally, absence or presence of relevant laws and policies focused on addressing non-communicable diseases (NCD) reflect the relative strength of preventive health policies in a country and thus may influence the income-obesity relationship. For instance, existing studies have found evidence of an association between smoking behavior and obesity incidence at both individual and population group levels [27–29]. That would suggest that laws meant to discourage smoking may influence obesity incidence.

We collected data on the macro-environmental variables from multiple sources including the WHO, the World Bank, International Telecommunications Union, UNESCO, International Energy Agency, and International Labor Organization [25, 30–34]. Our goal was to get data on multiple variables under each of the five major macro-environmental dimensions analyzed. Given the scope of our study in terms of 147 countries, this part of our country-specific data collection was particularly challenging. Nonetheless, we were able collect data on 21 underlying variables across the five major macro-environmental dimensions (see Table 1 below; also see S1 Table for data sources of these variables).

**Table 1:** Macro-environmental dimensions and respective variables.

Dimension	Variables
Globalization orientation	Social globalization index, Political globalization index, Mobile cellular subscription per 100 people, and Internet users per 100 people
Demographic characteristics	Age dependency ratio, Male literacy rate, Female literacy rate, Proportion of elderly (> = 65) population, Proportion of urban population, and Population density
Economic environment	Share of agriculture in the national GDP, Share of services in the national GDP, Access to electricity, Proportion of urban population with access to water, and Number of physicians per 1000 people
Labor market characteristics	Female labor force participation rate, Male labor force participation rate, Total labor force participation rate, and Proportion of females in the labor force
Strength of health policies	Law mandates that health warnings appear on tobacco packages, and Existence of an operational and multi-sectoral national NCD policy

### Statistical methods

Our empirical analysis used a hierarchical regression model with two stages [35]. In the first stage, we modelled the association between national income (GDPPC) and obesity prevalence. The country-specific estimates from the first stage of the model capture the changes in obesity prevalence in response to changes in GDPPC (i.e., income elasticities of obesity prevalence). In the second stage, we modelled the moderating effects of country-level macro-environmental variables on the income elasticity estimates from the first stage. Our model specification is as follows.

$${Obesity\_prev}_{c,t}=\alpha_{c}+\beta_{c}f({GDPPC}_{c,t})+\varepsilon_{c,t}\;\varepsilon_{c,t}∼N(0,{\sigma_{c,t}}^{2})$$

$$\left[\begin{array}{c} \alpha_{c}\\ \beta_{c} \end{array}\right]=\left[\begin{array}{c} X_{c}^{\prime }\;\theta_{\alpha }\\ X_{c}^{\prime }\;\theta_{\beta } \end{array}\right]+\left[\begin{array}{c} \pi_{\alpha ,c}\\ \pi_{\beta ,c} \end{array}\right]\;\left[\begin{array}{c} \pi_{\alpha ,c}\\ \pi_{\beta ,c} \end{array}\right]∼MVN(0,\Sigma )$$

*Obesity_prev_c,t_* refers to the obesity prevalence in a country *c* in year *t*. The key explanatory variable in the first stage is the per capita national income level of country c in the respective year *t* (*GDPPC_c,t_*). In the second stage multivariate regression model, country-specific intercepts (*α_c_*) and estimates of income-elasticities (*β_c_*) from the first stage were the dependent variables, and the independent variables were the country-level variables (*X_c_*) which represented the five dimensions of countries’ macro environments (globalization orientation, demographic characteristics, economic environment, labor market characteristics, and strength of health policies). The second stage coefficients *θ_β_* captured the moderating effects of these variables on the income elasticities of obesity prevalence. The variables (that are not dummy variables) in the second stage were standardized to enable us to compare their relative effects.

Given the debate about the pattern of relationship between obesity prevalence and national income [8,15–19], we specified a general functional form which could take one of the three possible forms–linear, quadratic, and log-log for the first stage regression model.

$Linear:\;{Obesity\_prev}_{c,t}=\alpha_{c}+\beta_{c}{GDPPC}_{c,t}$

${Quadratic:\;Obesity\_prev}_{c,t}=\alpha_{c}+\beta_{1c}{GDPPC}_{c,t}+\beta_{2c}{{GDPPC}_{c,t}}^{2}$

$Log‐log:\;ln({Obesity\_prev}_{c,t})=\alpha_{c}+\beta_{c}ln({GDPPC}_{c,t})$

We first determined the appropriate functional form that best captured the relationship between obesity prevalence and GDPPC in the data using model-free scatter plots as well as formal tests using relevant model fit indices. Specifically, we estimated separate models for the three functional forms and compared the model fits using the Mean Absolute Percentage Errors (MAPE).

For the second stage analysis, as noted earlier, we collected data on multiple variables to represent each of the five dimensions of countries’ macro-environments. However, all of these variables could not be included in the model because of the high correlations between them. Therefore, we followed an iterative forward-selection procedure and selected variables that have low correlation among themselves and improve model fit, ensuring that at least one variable is chosen from each dimension. Specifically, we started with a base second-stage regression model with no explanatory variables and added one variable at a time from the list of studied moderators. We assessed the model fit using Akaike Information Criterion (AIC) and Bayesian Information Criterion (BIC) and identified the most significant variable which had the best model fit to include as the moderator in the regression. We then excluded the dimension (e.g. globalization) of the moderator that has been selected, and selected another moderating variable from the other remaining dimensions using the same procedure as above. After at least one variable was chosen from each dimension, we repeated the procedure considering all remaining moderating variables.

After identifying the appropriate model specification and selecting the second stage moderators, we estimated three separate models with obesity prevalence among adult males, females and overall adult population as dependent variables. We estimated both stages of the model simultaneously as a Bayesian hierarchical model using Gibbs sampler, as this procedure is more efficient than estimating the two stages separately and provides unbiased goodness of fit measure [35]. Further, our two-stage model framework offers several advantages over a single stage model with interaction terms between national income and moderators. First, this framework allows us to estimate the income elasticity of obesity prevalence separately for each country. Secondly, while the data on obesity prevalence and national income were available for 40 years, data on country-level moderators were available only for recent years for the studied countries. The two-stage hierarchical model enabled us to take advantage of the extensive longitudinal data for estimating the income elasticity of obesity prevalence at individual country-level, and then study the moderating effects of macro-environment variables in a cross-sectional analysis. In addition, this model avoids multi-collinearity issue associated with multiple interaction terms in a one-stage model. The models were estimated using Bayesm package (rhierLinearModel) in R [36]. We ran 50,000 iterations for each model. We retained every 10^th^ draw to overcome the autocorrelation issue and used the first 30,000 iterations for burn-in. We assessed the convergence of our model using trace plots and Geweke convergence test for the second-level coefficients using Coda package in R [37]. We found that all of the coefficients (standard Z-scores) were insignificant, indicating the convergence of the model.

We forecasted the expected percentage change in adult obesity prevalence for the period 2019–2024 from IMF-projected national incomes and our analysis-based income elasticity estimates of obesity prevalence. Specifically, we made 1000 draws of income elasticity values from our model estimates for each country and calculated the percentage change in obesity level for a country as the drawn value of income elasticity multiplied by the percentage change in the IMF projected income of that country for each year in the forecast period. We then computed the average obesity growth and the associated prediction intervals across the draws. The obesity prevalence dataset (based on which the hierarchical model was estimated) in our study covered the period till 2014, and the IMF income projections data covered the period 2017–2024. For the intervening years (2015–2017), we used the actual growth rate in GDP from the World Bank for our forecasts.

## Results

### Obesity prevalence and trends

**Fig 1:**
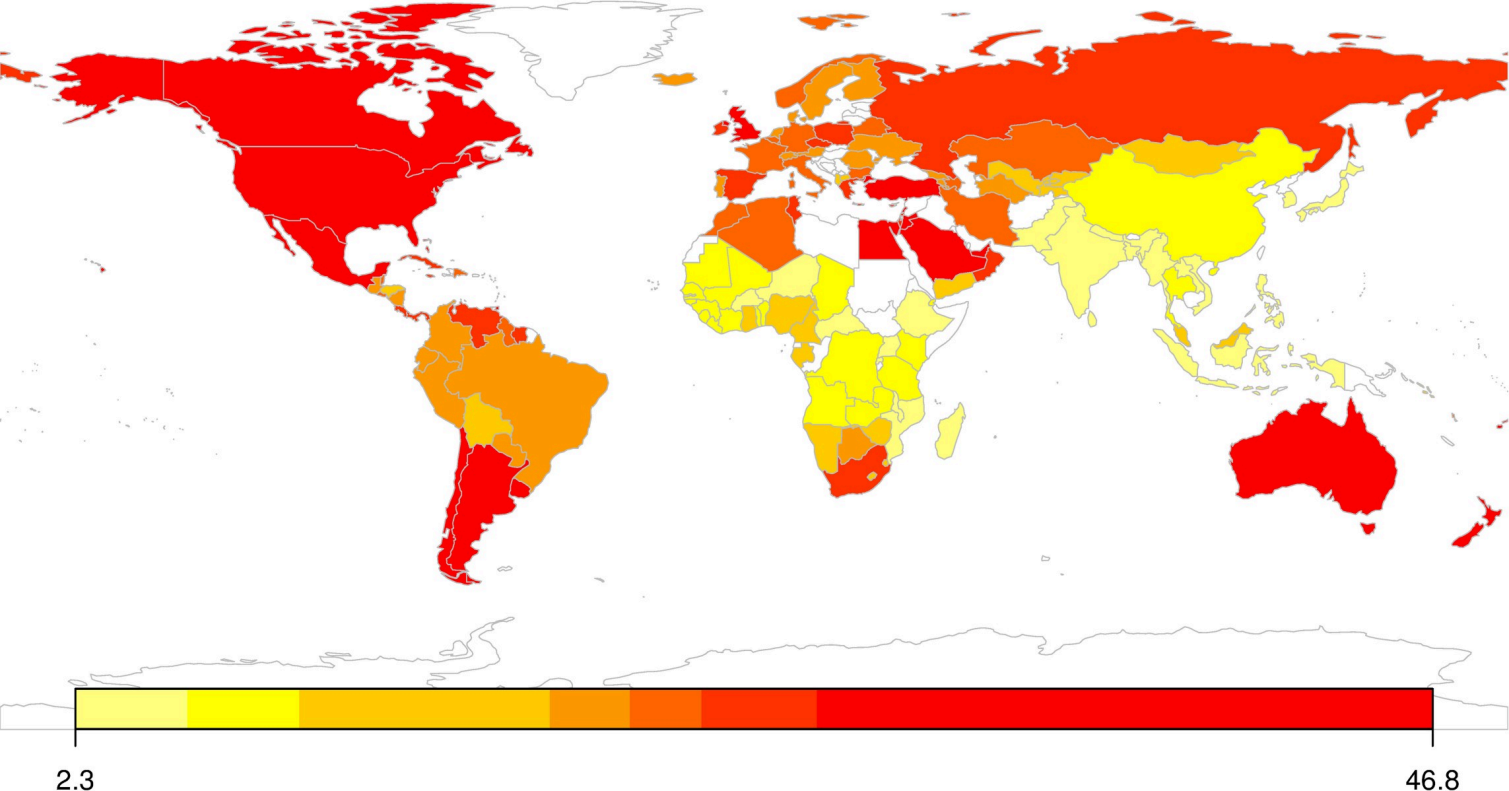
Prevalence of adult obesity in 147 countries worldwide. Proportion of obese adults in countries are represented by color scales. The map was created using Rworldmap package in R [38].

Across the studied countries, 13.6% of adult males and 20.9% of adult females had obesity in the year 2013. There was considerable variation in obesity prevalence across countries at different income levels and from different geographic regions as shown in Fig 1. To document the differential patterns in obesity prevalence across countries from different income and geographical groups, we used the World Bank’s classification of countries into four income groups (high, upper middle, lower middle and low income) and six regional/geographic groups (see S2 Table). We then calculated the average obesity prevalence across countries in respective groups (see S3 Table).

In terms of income groups, high income countries had the highest levels of adult obesity prevalence (22.72%) followed by upper middle-income countries (20.88%). Adult obesity prevalence was considerably lower among the lower middle-income countries (13.45%), but there was wide variation across countries in this group (standard deviation of 8.74). Low income countries had the lowest levels of obesity prevalence at 6.04% of adult population. Further, the gap between male and female obesity prevalence varied systematically across country groups. High income countries had comparable levels of obesity prevalence among adult males and females (21.2% vs. 24.5%), whereas lower-middle (8.99% vs. 17.87%) and low (2.35% vs. 9.65%) income countries had lower levels of male obesity prevalence than female obesity prevalence.

In terms of regions, North America had the highest levels of adult obesity prevalence (30.46%) while South Asia had the lowest (4.37%). Prevalence of obesity among adult females was markedly higher than male obesity prevalence in countries in South Asia (6.18 vs. 2.56), Sub-Saharan Africa (14.31 vs. 4.25), Middle East & North Africa (19.12% vs. 30.58%) and Latin America & Caribbean (17.2% vs. 28.59%). In terms of longitudinal trends, obesity prevalence among adults across the studied countries increased from 5.41% in 1975 to 17.51% in 2014 at a *Compounded Annual Growth Rate* (CAGR) of 3.06%. Adult obesity prevalence grew fastest in low-income countries (CAGR of 4.65%), and at the slowest rate in the high-income countries (CAGR of 2.69%).

### Longitudinal pattern of the income-obesity relationship

**Fig 2:**
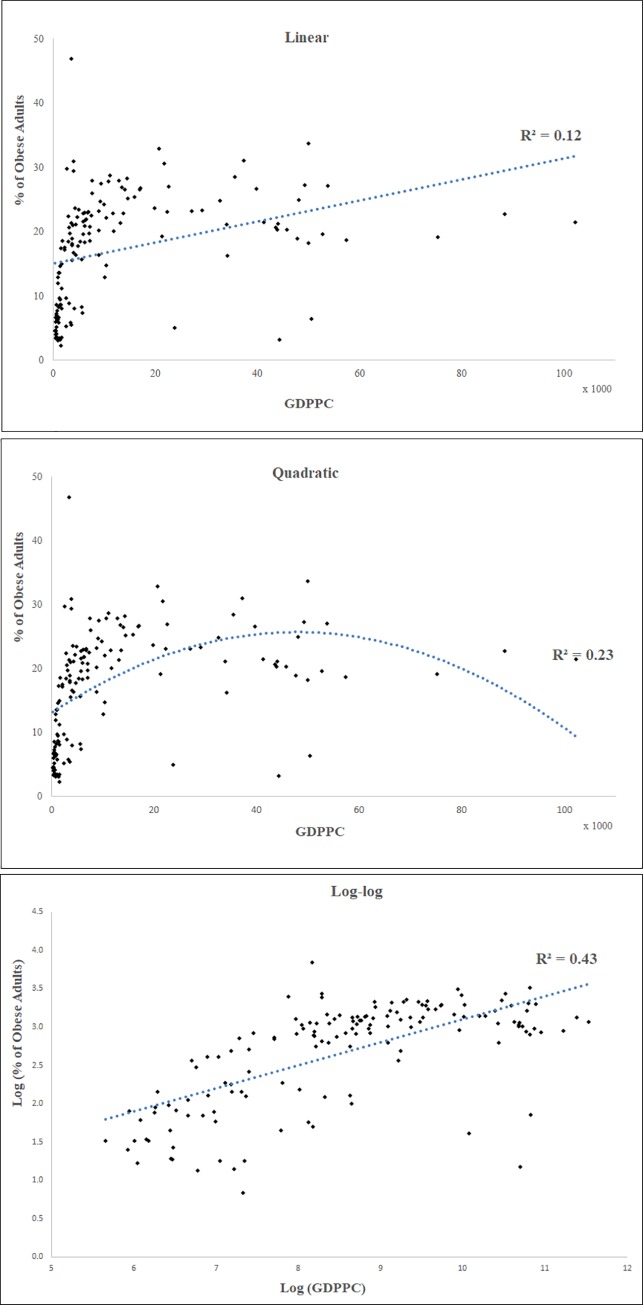
Relationship between obesity prevalence and national income (GDPPC). Trend lines are shown on the scatter plots of GDPPC vs. obesity prevalence along with R^2^ values separately for the three functional forms–linear, quadratic and log-log.

Fig 2 shows the scatter plots of adult obesity prevalence versus GDPPC across studied countries along with R^2^ values for the three functional forms (linear, quadratic, and log-log) for a sample year—2013. The plots show a positive non-monotonic relationship between GDPPC and obesity prevalence. In terms of formal tests, log-log model had the lowest MAPE value of 10.35 compared to 11.2 for linear model and 10.62 for quadratic model. Thus, the linear model had the worst fit, indicating that obesity prevalence did not increase proportionally with GDPPC. The empirical pattern was also inconsistent with an inverted-U shaped relationship, and thus, there was no support for the existence of an obesity Kuznets curve [8, 16]. The better fit of the log-log model indicated that the association between obesity prevalence and GDPPC was positive and stronger at lower income levels, but weakened (while still remaining positive) as national incomes increased. Therefore, we specified the hierarchical regression model in the log-log form for the remainder of our empirical analyses. A conceptually appealing upshot of the log-log model specification is that the coefficient estimate of log (GDPPC) represents the income elasticity of obesity prevalence, i.e., percentage change in obesity prevalence for a one-percentage change in GDPPC.

### Income elasticities of obesity prevalence

**Fig 3:**
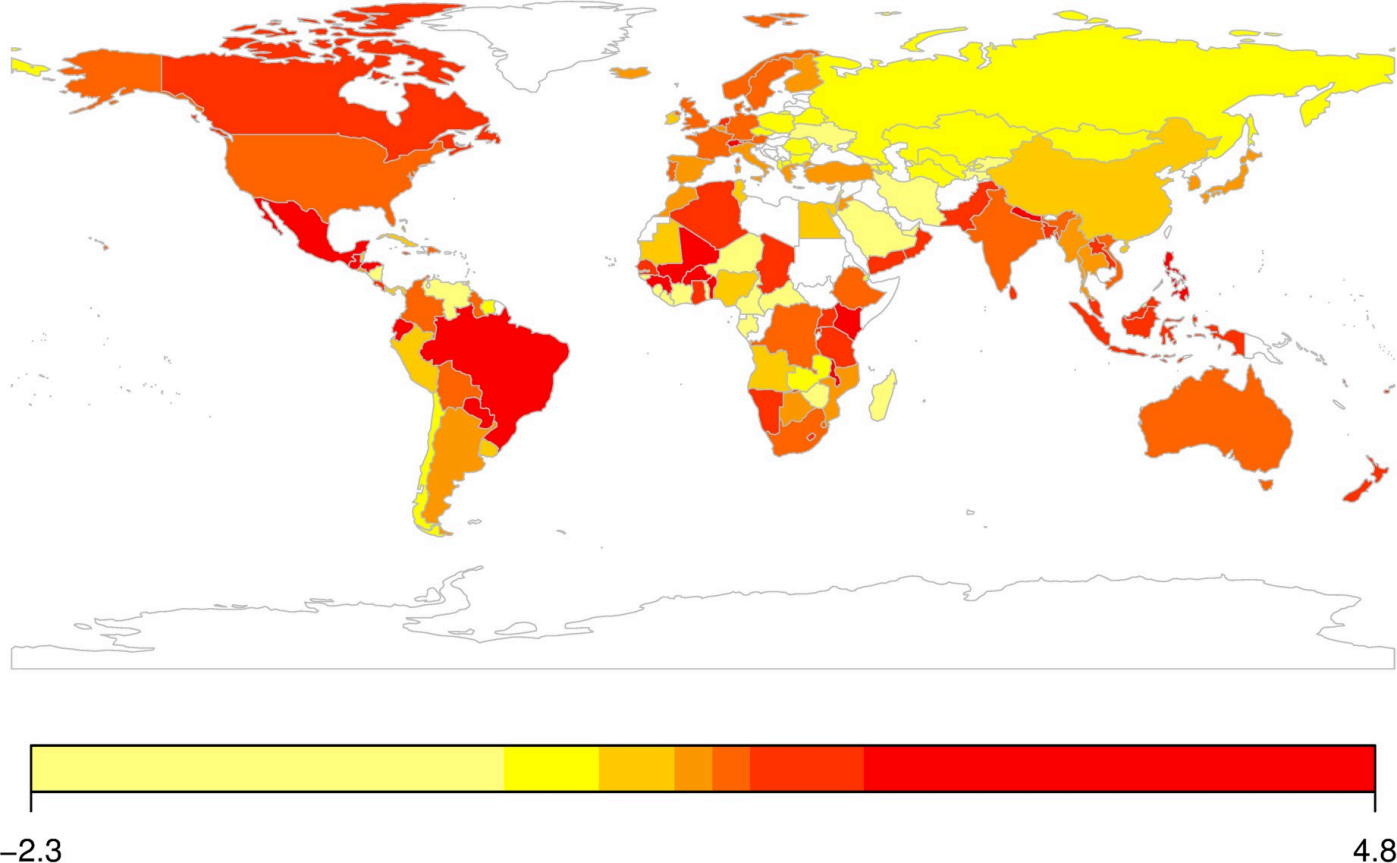
Distribution of estimated income elasticities of adult obesity prevalence across 147 countries. Income elasticities in countries are represented by color scales. The map was created using Rworldmap package in R [38].

The mean income elasticity estimates across countries were 1.23 (1.04, 1.42) for males and 1.01 (0.82, 1.18) for females. Thus, a 1% increase in GDPPC was on average associated with a 1.23% and a 1.01% increase in the prevalence of obesity among adult males and females, respectively. As shown in Fig 3, income elasticities varied widely across studied countries. A vast majority (82%) of the countries had positive elasticity values. Among those, some of the countries with the highest income elasticities for adult obesity prevalence were Kenya (3.4) in Africa, Nepal (2.49) in South Asia; Switzerland (2.36) in Europe; Canada (1.88) in North America; and Brazil (2.26) in Latin America. Countries with the lowest income elasticities were Ukraine (0.10), and Tajikistan (0.06). The few countries (e.g., Gabon, Togo, Central African Republic, Niger, Zimbabwe, Sierra Leone, and Liberia) that had negative associations between obesity prevalence and GDPPC are mostly from Africa and represent cases where even as national economic growth suffered, obesity prevalence grew.

### Macro-environmental moderators of income elasticities

Based on our iterative forward-selection procedure, the following macro-environmental variables were selected from each dimension: the political globalization index from the ‘globalization orientation’ dimension [10, 34]; the proportion of urban population from the ‘demographic characteristics’ dimension; the share of agriculture outputs in the national GDP from the ‘economic environment’ dimension; the percentage of females in the total labor force from the ‘labor market characteristics’ dimension, and the existence of law mandating health warnings on tobacco packages from the ‘strength of health policies’ dimension. These variables can be interpreted as proxies for characterizing the nature of the respective macro-environmental dimensions in a particular country.

As evident from Table 2, statistically significant estimates of the second stage of the hierarchical regression model show that the association between national income and obesity prevalence was moderated by political globalization index, urbanization and share of agricultural output in the national GDP. In particular, the positive moderating effect of political globalization index indicates that income elasticities were higher for countries that scored high in the political globalization index. On the other hand, urbanization had a negative effect on income elasticities indicating that income elasticities were lower for more urbanized countries. Further, as shown in Table 2, income elasticities were lower in countries with higher share of agriculture in the national GDP. In terms of the relative magnitudes of the moderating effects, the effect of urbanization was the strongest (-0.391), followed by those of political globalization index (0.326) and agriculture’s share in the national GDP (-0.295).

**Table 2:** Moderating effects of macro-environmental variables on the national income elasticity of population obesity prevalence^1^.

Macro-environmental variables	Estimates (95% credible interval)		
	*Male obesity prevalence*	*Female obesity prevalence*	*Overall obesity prevalence*
Intercept	1.546 (1.000, 2.092)	1.193 (.654, 1.732)	1.309 (.768, 1.850)
Political Globalization index	.319 (.110, .529)	.310 (.106, .514)	.326 (.121, .531)
Urbanization	-.395 (-.637, -.154)	-.394 (-.630, -.158)	-.391 (-.629, -.153)
Share of agriculture in the national GDP	-.411 (-.638, -.183)	-.246 (-.467, -.024)	-.295 (-.517, -.072)
Percentage of females in the total labor force	.011 (-.172, .194)	.026 (-.150, .201)	.028 (-.150, .206)
Existence of law mandating health warnings on tobacco packages	-.363 (-.960, .234)	-.216 (-.803, .371)	-.252 (-.840, .337)

^1^A statistically significant (i.e., credible interval does not include zero) positive (or, negative) coefficient estimate of a moderating variable in this table indicates that the income elasticity increases (or, decreases) with that variable

### Forecasted trends in obesity prevalence

**Fig 4:**
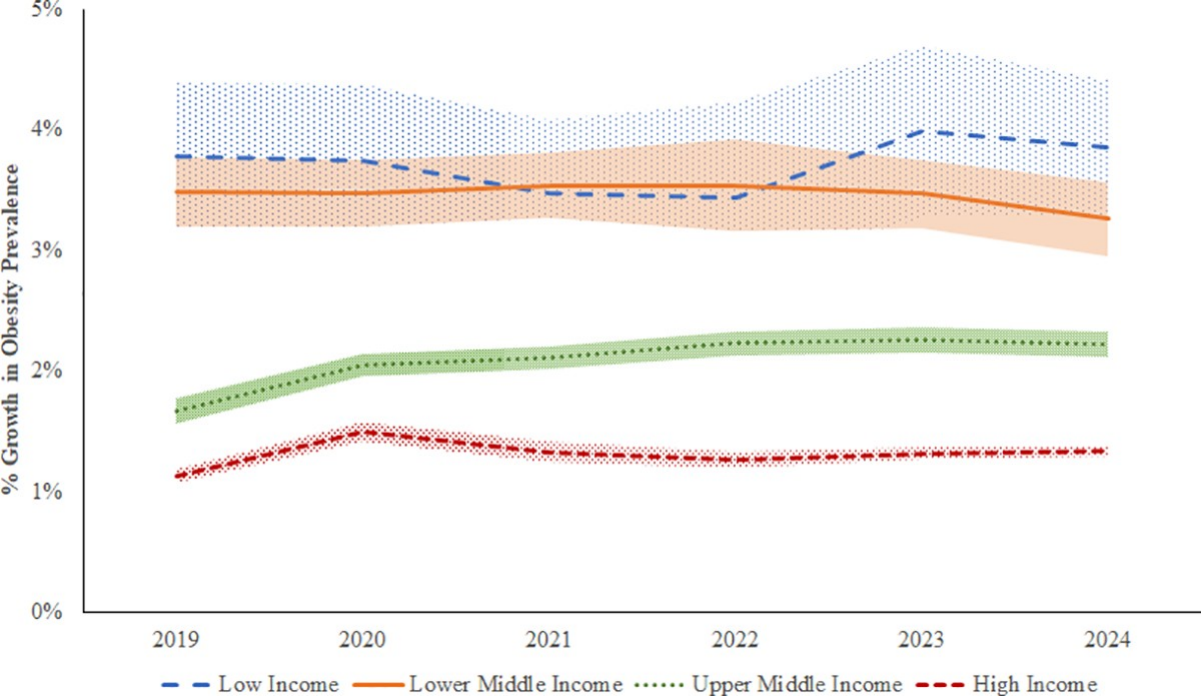
Forecasted percentage growth in adult obesity prevalence for countries grouped by national income-level.

We used the estimated income elasticities from our hierarchical model and the projected national incomes from the IMF to forecast annual growth rates in adult obesity prevalence for the years 2019–2024 (see S4 Table). Our forecasts showed that the average adult obesity prevalence across the studied countries would increase from 17.7% in 2014 to 21.6% in 2024. Fig 4 shows the forecasted average annual growth rates in obesity prevalence by the World Bank classification of countries based on their national income-level (i.e., high-income, upper middle-income, lower middle-income, and low-income). Obesity prevalence would increase at average annual rates of 1.32%, 2.09%, 3.46% and 3.72% among high-income, upper middle-income, lower middle-income and low-income countries, respectively.

**Fig 5:**
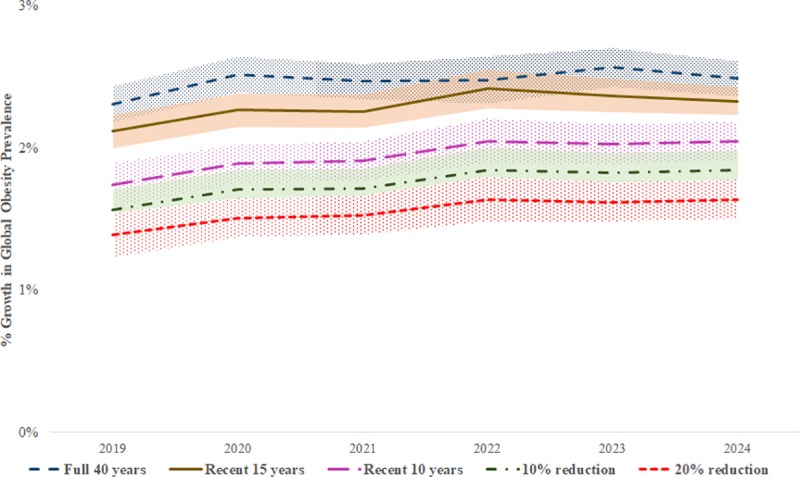
Sensitivity analysis: Forecasted percentage growth in adult obesity prevalence across 147 countries under five different scenarios. Five scenarios shown in the figure are income elasticities estimated using (a) full 40 years of data (b) recent 15 years of data (c) recent 10 years of data, (d) 10% lower elasticities than the estimates using recent 10 years of data, and (e) 20% lower elasticities than the estimates using recent 10 years of data.

Our forecasts assumed that income elasticities would remain constant at their estimated levels during the study period. To assess the sensitivity of our forecasts to this assumption, we conducted sensitivity analysis with respect to potential temporal variation in income elasticity estimates in several ways. First, to capture any changes in elasticities in the recent past, we conducted sensitivity analysis with income elasticity estimates from the most recent 15 and 10 years of data (as opposed to the full 40 years of data in our main analysis). Second, to capture any changes in elasticities over future time periods, we further forecasted future obesity prevalence under two potential “what if” scenarios–if the elasticities decline (due to policy actions) over the forecasted period (2019–2024) for a total drop of 10% and 20% from their values estimated from the most recent 10 years of data. Forecasted trends in obesity prevalence under these different scenarios are shown in Fig 5.

Our forecasts showed that, obesity prevalence would continue to grow during the period 2019–2024 at an average annual rate of 2.47% based on elasticities estimated with the full 40 years of data compared to average annual rates of 2.3% and 1.95% based on elasticities estimated with recent 15 and 10 years of data respectively. If income elasticities were to decrease by 10% as a result of policy actions, then the average annual growth rate in obesity prevalence during 2019–2024 would reduce to 1.76%. Under the more optimistic scenario of income elasticities reducing by 20% during the forecast period, obesity prevalence would grow at an average annual rate of 1.56%. Thus, taken together, our forecasts showed that the average annual growth rate of obesity prevalence across the studied countries would range from 1.56% to 2.47% during the forecast period (2019–2024) under a variety of empirically driven income elasticity estimate scenarios.

## Discussion

In this study, using an extensive dataset covering 147 countries over a period of 40 years, we systematically investigated the pattern and strength of the relationship between population obesity prevalence and national income, as well as the macro-environmental moderators of this relationship. We found that population obesity prevalence has a positive relationship with national income but no evidence that the relationship, while weakening, actually turns negative at higher income levels. This finding is in contrast to the conjectured existence of an obesity Kuznets curve in some studies [8, 16]. In terms of the strength of the income-obesity relationship across our analyzed countries, we find that a 1% increase in per capita income was on average associated with a 1.23% and a 1.01% increase in the prevalence of obesity among adult males and females, respectively.

As for macro-environmental moderators, we found that the income elasticity of obesity prevalence in a country increases with the extent of its political globalization, but decreases with higher proportion of agricultural outputs in its national GDP and with higher proportion of its population living in urban areas. We should note here that while our analysis provides statistical estimates of the directional effects of the selected macro-environmental moderators on income elasticity, it does not shed any direct insight into why we find such directional effects. However, drawing on the findings from the existing literature, we discuss below potential reasons behind the observed directional effects of the three moderators noted earlier.

Our finding that the positive relationship between national income and population obesity prevalence gets stronger for countries that score higher in the political globalization index is consistent with past studies [10, 34], which found a positive association between a country’s population obesity prevalence and the extent of its political globalization. The argument here is that political factors relating to the formation of regional trade blocks, or participation in various international treaties, may play a role, by acting as a precursor to greater economic integration via the opening of food markets to free trade and consequent nutritional change associated with overweight [10]. Our finding that a higher share of agricultural output of a country in its GDP attenuates the positive relationship between national income and population obesity prevalence is also consistent with the well accepted upshots of increased industrialization of a country’s economy on its food sector [39]. The rationale here is that as a country’s economy shifts from being a more agrarian one to a more industrialized one, its food sector goes through a concomitant shift into more processed and fast foods linked to higher population obesity incidence [40].

While our finding that a higher proportion of a country’s population living in urban areas attenuates the positive relationship between national income and population obesity prevalence may seem counterintuitive at first glance, it is in fact consistent with recent findings that BMI is increasing at the same rate or faster in rural areas than in cities, particularly in low- and middle-income countries [41–43]. These findings are attributed to the ‘urbanization of rural life’ phenomenon [41]. Rural areas, which have historically had lower food consumption due to their lower incomes and higher energy expenditure in the daily work and domestic activities, had lower BMI traditionally. However, with increasing incomes and food availability (e.g. through increased distribution of foods), rural areas have been witnessing disproportionately more spending on food, and a concomitant reduction in energy expenditure due to increasing mechanization of agriculture and redundancy of some traditional household tasks [42].

As for our forecasts of future global trends in adult obesity levels, based on IMF’s latest income growth projections for the years 2019–2024, they indicate that obesity prevalence is likely to grow at an average annual rate of 2.47% across the studied countries. More importantly, the proportion of obese adults would exceed 20% of the adult population in the studied countries by 2021, much earlier than 2030 as suggested in the literature [44]. However, the only past study that reported yearly forecasts of obesity prevalence at the global level, did so based on existing trends in obesity growth rates [44]. In contrast, we forecast obesity growth rates based on IMF’s projected growths in national income levels, which have been shown to be critical correlates of national trends in obesity prevalence. Taken together, our findings echo the conclusions from previous research [1] that if current trends continue, then UN NCD targets are unlikely to be met.

A key implication of our findings is that the national income level of a country continues to have a positive relationship with the prevalence of population obesity, even at quite high-income levels. As most people currently live in low- and middle-income countries with rising incomes, our findings underscore the urgent societal imperatives for effective policy initiatives, especially those that target the concomitant “nutrition transition” process with economic affluence, to break or at least further weaken the positive relationship of population obesity prevalence with national income. Such societal imperatives align with the calls for a shift away from the dominant development paradigm that focuses almost exclusively on economic growth to a paradigm of sustainable development [45]. The practical challenge of sustainable development, of course, is how we can enjoy economic growth without adversely impacting our natural environment and personal health and well-being.

In the specific context of our study, the critical challenge essentially becomes how we can leverage rising income levels in a country in managing its concomitant nutrition transition process to break or at least weaken the positive association between income growth and obesity prevalence. As one would expect, it will require a concerted policy-driven effort on multiple aspects of current socio-economic system [46]. In that context, the WHO Global NCD Action Plan [47], ECHO report [48] and other expert opinions [49–52] outline a number of well-intended policy recommendations—such as, development of national food and nutrition policies and action plans, implementation of food taxes and subsidies that incentivize healthy consumption behaviors, restricting children’s exposure to marketing of unhealthy foods, and public awareness programs on diet and physical activity. Moving forward, an important direction for future research needs to be on investigating how the aforesaid policy recommendations can best work in practice individually and in synergistic combinations.

In conclusion, given the highly significant health and economic costs of obesity and the clear importance of economic development, it is vital to gain an in-depth and systematic understanding into the association between obesity prevalence and national income. Using a unique dataset in terms of countries and time period analyzed compared to any of the extant studies, our study offers hitherto unexplored insights into the pattern and strength of the income-obesity relationship, as well as into the roles of macro-environmental moderators of this relationship. Taken together, we strongly believe that our study advances the current understanding of the relationship between national income and obesity prevalence in novel and substantive ways.

At the same time, we also recognize that our study is not without limitations for future research to address. Specifically, our aggregate country-level data and analysis do not allow us to consider the patterns of obesity prevalence across population sub-groups within a country [10]. Also, our dependent variable–obesity prevalence—is based on a dichotomous classification of individuals in populations as obese/non-obese based on a BMI cut-off point (BMI > 30). Though using this cut-off point has the appeal of more precisely capturing the increase in weight that conveys the most important change in risk (the transition from normal to overweight or obesity), this measure is not sensitive to further increases in weight among those already obese. Finally, data constraints limited us from studying other interesting potential moderators of income elasticities of obesity prevalence. For example, some studies [53,54] have found association between income inequality and obesity incidence in populations. Investigating how income inequality specifically moderates the relationship between national income level and population obesity prevalence would be an important avenue for future research.

## Supporting information

S1 Table Variables and their sources. (DOCX)

S2 Table Countries by income groups. (DOCX)

S3 Table Obesity prevalence across countries by income group and region. (DOCX)

S4 Table Projected trends in obesity prevalence. (DOCX)
