# Supplementary material for: The association between national income and adult obesity prevalence: Empirical insights into temporal patterns and moderators of the association using 40 years of data across 147 countries
Source: PLoS One. 2020 May 13;15(5):e0232236. doi: 10.1371/journal.pone.0232236 (PMC7219711; doi:10.1371/journal.pone.0232236)
Supplement: S1 Table — (DOCX) [file pone.0232236.s001.docx]

# **S1 Table.** Variables and their sources

| **Variable** | **Description** | **Years^a^** | **Sources** | **Mean** | **SD** |
| --- | --- | --- | --- | --- | --- |
| Male_obesity^b^ | Proportion of adult males with BMI > 30 | 1975-2014 | WHO Global Health Observatory database^1^ | 13.63 | 8.61 |
| Female_obesity^b^ | Proportion of adult females with BMI > 30 | 1975-2014 | WHO Global Health Observatory database^1^ | 20.93 | 9.76 |
| Overall_obesity^c^ | Proportion of adults with BMI > 30 | 1975-2014 | WHO Global Health Observatory database^1^ | 17.27 | 8.68 |
| GDPPC | GDP Per Capita in Constant 2010 US Dollars | 1975-2014 | WB Development Indicators database^2^ | 13,309 | 18,535 |
| **Globalization orientation** | | | | | |
| SGLOBAL | Social globalization index | 2010 | KOF Index of Globalization^3,4^ | 47.81 | 21.19 |
| PGLOBAL | Political globalization index | 2010 | KOF Index of Globalization^3,4^ | 68.34 | 20.18 |
| MOBILE | Mobile cellular subscriptions (per 100 people) | 2005-15 | World Telecommunication/  ICT Development Report and database^2,5,d^ | 79.11 | 36.19 |
| INTNET | Internet users (per 100 people) | 2005-15 | World Telecommunication/  ICT Development Report and database^2,5,d^ | 31.62 | 25.94 |
| **Demographic characteristics** | | | | | |
| AGEDEP | Age dependency ratio (% of working-age population) | 2005-15 | WB Development Indicators database^2^ | 61.33 | 18.07 |
| FEMLIT | Literacy rate, adult female (% of females) | 2005-15 | UNESCO Institute for Statistics^2,6, d^ | 81.37 | 22.44 |
| MALELIT | Literacy rate, adult male (% of males) | 2005-15 | UNESCO Institute for Statistics^2,6, d^ | 87.25 | 15.34 |
| OLD65 | Population ages 65 and above (% of total) | 2010 | WB Development Indicators database^2^ | 7.47 | 5.11 |
| URBAN | Urban population (% of total) | 2010 | WB Development Indicators database^2^ | 55.09 | 23.34 |
| PDENSITY | Population density (people per sq. km of land area) | 2010 | WB Development Indicators database^2^ | 181.63 | 601.84 |
| **Economic environment** | | | | | |
| AGRISH | Agriculture, value added (% of GDP) | 2005-14 | WB Development Indicators database^2^ | 13.61 | 13.17 |
| SERVSH | Services, value added (% of GDP) | 2005-15 | WB Development Indicators database^2^ | 56.68 | 14.04 |
| ELECACCES | Access to electricity (% of population) | 2005-15 | Sustainable Energy for ALL (SE4ALL) Global Electrification database^2,7, d^ | 76.42 | 31.34 |
| WATER | Improved water source (% of urban population with access) | 2010 | WB Development Indicators database^2^ | 94.43 | 7.46 |
| DOCTOR | Physicians (per 1,000 people) | 2008-12 | WHO Global Health Observatory Database^1^ | 1.49 | 1.46 |
| **Labor market characteristics** | | | | | |
| FLABPART | Labor force participation rate, female | 2005-15 | ILO Key Indicators of the Labour Market database^2,8, d^ | 53.81 | 15.81 |
| MLABPART | Labor force participation rate, male | 2005-15 | ILO Key Indicators of the Labour Market database^2,8, d^ | 75.47 | 7.43 |
| TLABPART | Labor force participation rate, total | 2005-15 | ILO Key Indicators of the Labour Market database^2,8, d^ | 64.72 | 9.93 |
| FLABPCT | Labor force, female (% of total labor force) | 2005-15 | ILO Key Indicators of the Labour Market database^2,8, d^ | 41.30 | 8.62 |
| **Strength of health policies** | | | | | |
| TOBPOL | Law mandates that health warnings appear on tobacco packages | 2010 | WHO Global Health Observatory Database^1^ |  |  |
| NCDPOL | Existence of an operational, multi-sectoral national NCD policy | 2013-15 | WHO Global Health Observatory Database^1^ |  |  |

**^a^** Year/s for which data on a variable was collected. For the obesity prevalence and GDPPC (first-stage analysis), we use yearly data over the analysis period of 40 years (1975-2014). For the macro-environmental variables (second-stage analysis), we use their country-specific average values over the available years of data.

**^b^** Obesity prevalence data in the WHO indicators database has been compiled from population-based surveys by the NCD Risk Factor Collaboration^9^.

**^c^** Overall population obesity is calculated from male and female obesity levels and the population shares of the respective genders.

**^d^** These variables are also available from the World Bank Development Indicators Database.
