# Supplementary material for: The association between national income and adult obesity prevalence: Empirical insights into temporal patterns and moderators of the association using 40 years of data across 147 countries
Source: PLoS One. 2020 May 13;15(5):e0232236. doi: 10.1371/journal.pone.0232236 (PMC7219711; doi:10.1371/journal.pone.0232236)
Supplement: S2 Table — (DOCX) [file pone.0232236.s002.docx]

# **S2 Table.** Countries by income groups

| **Income group** | **Countries** | |
| --- | --- | --- |
| **High income** | | Antigua and Barbuda, Australia, Austria, Bahamas, Barbados, Belgium, Brunei Darussalam, Canada, Chile, Cyprus, Czech Republic, Denmark, Finland, France, Germany, Greece, Iceland, Ireland, Israel, Italy, Japan, Republic of Korea, Luxembourg, Malta, Netherlands, New Zealand, Norway, Oman, Poland, Portugal, Saudi Arabia, Seychelles, Singapore, Spain, St. Kitts and Nevis, Sweden, Switzerland, Trinidad and Tobago, United Arab Emirates, United Kingdom, United States, Uruguay |
| **Upper middle income** | | Albania, Algeria, Angola, Argentina, Azerbaijan, Belarus, Belize, Botswana, Brazil, Bulgaria, China, Colombia, Costa Rica, Cuba, Dominica, Dominican Republic, Ecuador, Fiji, Gabon, Georgia, Grenada, Guyana, Islamic Republic of Iran, Jamaica, Jordan, Kazakhstan, Lebanon, Macedonia, Malaysia, Mauritius, Mexico, Namibia, Panama, Paraguay, Peru, Romania, Russian Federation, South Africa, St. Lucia, St. Vincent and the Grenadines, Suriname, Thailand, Turkey, Turkmenistan, Venezuela |
| **Lower middle income** | | Armenia, Bangladesh, Bolivia, Cameroon, Congo, Cote d'Ivoire, Djibouti, Egypt, El Salvador, Ghana, Guatemala, Honduras, India, Indonesia, Kenya, Kyrgyz Republic, Lao PDR, Lesotho, Mauritania, Mongolia, Morocco, Myanmar, Nicaragua, Nigeria, Pakistan, Philippines, Solomon Islands, Sri Lanka, Swaziland, Tajikistan, Tonga, Tunisia, Ukraine, Uzbekistan, Vanuatu, Vietnam, Yemen, Rep., Zambia |
| **Low income** | | Benin, Burkina Faso, Central African Republic, Chad, Ethiopia, Gambia, Guinea, Guinea-Bissau, Liberia, Madagascar, Malawi, Mali, Mozambique, Nepal, Niger, Rwanda, Senegal, Sierra Leone, Tanzania, Togo, Uganda, Zimbabwe |
