# Supplementary material for: The association between national income and adult obesity prevalence: Empirical insights into temporal patterns and moderators of the association using 40 years of data across 147 countries
Source: PLoS One. 2020 May 13;15(5):e0232236. doi: 10.1371/journal.pone.0232236 (PMC7219711; doi:10.1371/journal.pone.0232236)
Supplement: S3 Table — (DOCX) [file pone.0232236.s003.docx]

# **S3 Table.** Obesity prevalence across countries by income group and region

| **Country group** | | **N** | **Male obesity prevalence** | | **Female obesity prevalence** | | **Overall adult obesity prevalence** | | **GDPPC** | |
| --- | --- | --- | --- | --- | --- | --- | --- | --- | --- | --- |
|  |  |  | **Mean** | **SD** | **Mean** | **SD** | **Mean** | **SD** | **Mean** | **SD** |
| Total | 147 | | 13.63 | 8.61 | 20.93 | 9.76 | 17.27 | 8.68 | 13308 | 18535 |
| **Countries by income group** | | | | | | | | | | |
| High | 42 | | 21.18 | 5.82 | 24.47 | 8.44 | 22.72 | 6.46 | 36684 | 20289 |
| Upper middle | 45 | | 16.02 | 4.97 | 25.71 | 6.75 | 20.88 | 5.24 | 7097 | 2610 |
| Lower middle | 38 | | 8.99 | 7.48 | 17.87 | 10.39 | 13.45 | 8.74 | 2190 | 1034 |
| Low | 22 | | 2.35 | 0.87 | 9.65 | 3.67 | 6.04 | 2.19 | 591 | 186 |
| **Countries by region** | | | | | | | | | | |
| East Asia & Pacific | 19 | | 11.82 | 10.83 | 16.26 | 13.59 | 14.02 | 12.10 | 15208 | 18616 |
| Europe & Central Asia | 37 | | 19.67 | 3.78 | 21.28 | 3.81 | 20.50 | 3.27 | 28041 | 25860 |
| Latin America & Caribbean | 32 | | 17.20 | 3.88 | 28.59 | 3.96 | 22.95 | 3.61 | 8447 | 4974 |
| Middle East & North Africa | 14 | | 19.12 | 6.93 | 30.58 | 8.07 | 24.46 | 6.78 | 11759 | 12103 |
| North America | 2 | | 29.55 | 4.74 | 31.35 | 4.31 | 30.46 | 4.53 | 49585 | 503 |
| South Asia | 5 | | 2.56 | 0.90 | 6.18 | 1.86 | 4.37 | 1.35 | 1508.98 | 1093.71 |
| Sub-Saharan Africa | 38 | | 4.25 | 3.17 | 14.31 | 7.58 | 9.31 | 5.28 | 2321.18 | 3136.18 |
