# Supplementary material for: The association between national income and adult obesity prevalence: Empirical insights into temporal patterns and moderators of the association using 40 years of data across 147 countries
Source: PLoS One. 2020 May 13;15(5):e0232236. doi: 10.1371/journal.pone.0232236 (PMC7219711; doi:10.1371/journal.pone.0232236)
Supplement: S4 Table — (DOCX) [file pone.0232236.s004.docx]

# **S4 Table.** Projected trends in obesity prevalence

|  | **% Growth in Obesity Prevalence (95% prediction intervals)** | | | | | | | | |  |  |
| --- | --- | --- | --- | --- | --- | --- | --- | --- | --- | --- | --- |
| **Country group** | | | **2019** | **2020** | | **2021** | **2022** | | **2023** | | **2024** |
| High Income | | 1.13%  (1.07%, 1.19%) | | 1.50%  (1.42%, 1.58%) | 1.33%  (1.25%, 1.41%) | | | 1.27%  (1.2%, 1.34%) | 1.32%  (1.27%, 1.37%) | | 1.34%  (1.29%, 1.39%) |
| Upper Middle Income | | 1.67%  (1.56%, 1.78%) | | 2.05%  (1.96%, 2.14%) | 2.11%  (2.02%, 2.20%) | | | 2.23%  (2.13%, 2.33%) | 2.26%  (2.15%, 2.37%) | | 2.22%  (2.12%, 2.32%) |
| Lower Middle Income | | 3.49%  (3.20%, 3.78%) | | 3.47%  (3.19%, 3.75%) | 3.54%  (3.27%, 3.81%) | | | 3.54%  (3.16%, 3.92%) | 3.47%  (3.19%, 3.75%) | | 3.26%  (2.96%, 3.56%) |
| Low Income | | 3.78%  (3.17%, 4.39%) | | 3.75%  (3.13%, 4.37%) | 3.47%  (2.86%, 4.08%) | | | 3.44%  (2.65%, 4.23%) | 3.99%  (3.29%, 4.69%) | | 3.86%  (3.30%, 4.42%) |
